# Artificial Intelligence Software to Accelerate Screening for Living Systematic Reviews

**DOI:** 10.1007/s10567-025-00519-5

**Published:** 2025-04-18

**Authors:** Matthew Fuller-Tyszkiewicz, Allan Jones, Rajesh Vasa, Jacqui A. Macdonald, Camille Deane, Delyth Samuel, Tracy Evans-Whipp, Craig A. Olsson

**Affiliations:** 1 School of Psychology, Faculty of Health, Deakin University, Geelong, Australia; 2 SEED Centre for Lifespan Research, Deakin University, 221 Burwood Highway, Burwood, Melbourne, VIC Australia; 3 Applied Artificial Intelligence Institute, Deakin University, Melbourne, Australia; 4 Faculty of Health, Deakin University, Melbourne, Australia

**Keywords:** Systematic reviews, Artificial intelligence, Machine learning, Efficiency, Accuracy

## Abstract

Systematic and meta-analytic reviews provide gold-standard evidence but are static and outdate quickly. Here we provide performance data on a new software platform, LitQuest, that uses artificial intelligence technologies to (1) accelerate screening of titles and abstracts from library literature searches, and (2) provide a software solution for enabling living systematic reviews by maintaining a saved AI algorithm for updated searches. Performance testing was based on LitQuest data from seven systematic reviews. LitQuest *efficiency* was estimated as the proportion (%) of the total yield of an initial literature search (titles/abstracts) that needed human screening prior to reaching the in-built stop threshold. LitQuest algorithm *performance* was measured as work saved over sampling (WSS) for a certain recall. LitQuest *accuracy* was estimated as the proportion of incorrectly classified papers in the rejected pool, as determined by two independent human raters. On average, around 36% of the total yield of a literature search needed to be human screened prior to reaching the stop-point. However, this ranged from 22 to 53% depending on the complexity of language structure across papers included in specific reviews. Accuracy was 99% at an interrater reliability of 95%, and 0% of titles/abstracts were incorrectly assigned. Findings suggest that LitQuest can be a cost-effective and time-efficient solution to supporting living systematic reviews, particularly for rapidly developing areas of science. Further development of LitQuest is planned, including facilitated full-text data extraction and community-of-practice access to living systematic review findings.

## Introduction

In the social and medical sciences, systematic and meta-analytic reviews are considered the gold standard of evidence (Aromataris et al., 2024; Higgins et al., 2023). These reviews enable a comprehensive and representative snapshot of current evidence for a given research topic, designed to reduce biases inherent in selective searches and managing uncertainties when results vary between studies (Page et al., 2021). In recognition of these benefits, systematic reviews have become increasingly common, with one study estimating that the number of systematic review and meta-analyses published from 1995 to 2017 increased by more than 4000% (Niforatos et al., 2019).

**Fig. 1:**
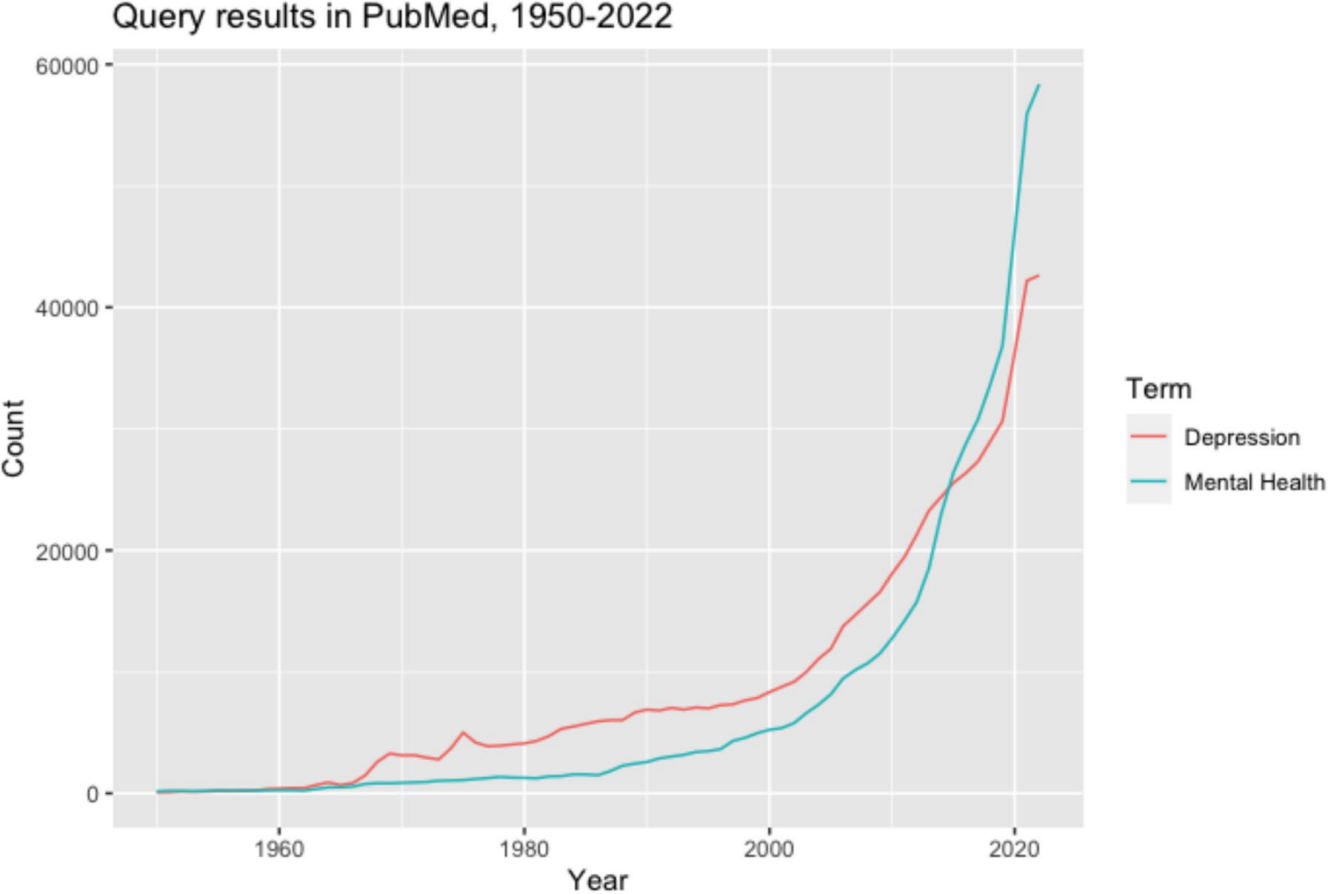
Illustration of exponential growth in mental health literature via PubMed search

However, systematic reviews are labour intensive; it is estimated that the average, human-led review takes 67 weeks from registration to publication (Borah et al., 2017), and that at least one-quarter of reviews may require updating within 1–2 years of publication (Shojania et al., 2007). This raises important questions about the viability of best-practice, human-led, reviews in the context of an exponentially increasing scientific literature (as illustrated in Fig. 1). Indeed, in some fields, maintaining a comprehensive review is already infeasible (Shemilt et al., 2014), with the problem of obsolescence of reviews only likely to extend broadly across fields in the future.

To address challenges to the production of high-quality, up-to-date reviews, new computer-assisted approaches are being developed with the specific aim of expediting production and reducing human error in the review process. This assistance has ranged from automated detection and deletion of duplicate records within a literature search (Adil et al., 2019; Elmagarmid et al., 2006), to search-query assistance to ensure that searches yield relevant literature (Grames et al., 2019), to artificial intelligence (AI) approaches to rank article relevance to expedite the screening process (Tsou et al., 2020), AI to extract relevant text passages and data for risk of bias assessment (Soboczenski et al., 2019), and literature synthesis and data summarisation (Marshall et al., 2020). Automation has also been used to support continually updated reviews (so-called ‘Living Systematic Reviews’) to prevent existing reviews from becoming outdated (Elliot et al., 2014).

Some of the more prominent automation tools (e.g. ASReview, Abstrackr, RobotAnalyst) focus on AI-assisted article screening in recognition that paper screening and selection is one of the most time-consuming elements of a review (Carver et al., 2013). Empirical evaluation of these AI-assisted review tools has been typically based on a common evaluation approach where researchers evaluate the proportion of articles in a search that did not need to be manually screened (due to AI screening) to achieve a specified level of accuracy in retrieving the relevant articles in the search pile. This evaluation approach is often referred to as work saved over sampling (WSS; Cohen et al., 2006), and the target proportion of relevant articles to retrieve is often set at 95% of total relevant articles within the full set of articles from one’s database search. Such studies have shown averaged estimates of up to 80% time saving (i.e. only having to screen 20% of total search results to retrieve 95% of relevant articles) in the screening phase from using an AI approach relative to manual screening of all articles (Rathbone et al., 2015; Tsou et al., 2020; van de Schoot et al., 2021). Van de Schoot and colleagues showed that ASReview can detect 70–100% of relevant articles from their test datasets based on manual screening of 10% of the total number of to-be-screened article abstracts. Further, Gates et al. (2019) showed that an AI-assisted review can pick up many relevant articles that are missed by a single human screener.

Beyond screening, there has been a proliferation of Generative AI-based applications to other aspects of reviewing global literature (Khalil et al., 2022). This new breed of applications (e.g. Elicit, Scite.ai) leverage large language models in surfacing relevant supporting literature given a lay query related to a research question, providing concise text summaries of both individual articles and across the base of literature for a topic, and providing some capability towards automated data extraction. However, while offering productivity in navigating and consuming literature on a given topic or question, questions remain about how rigorous and extensive their coverage of relevant literature is (Whitfield & Hofmann, 2023; Yao et al., 2024). Relatedly, the tools face challenges in the form of licensing and usage constraints for academic literature that is not open access.

Here we describe the performance of the first component of a larger Living Review System (LitQuest, previously referred to as the Living Knowledge System; Grbin et al., 2022), which is a new artificial intelligence-based platform that our team has developed to accelerate screening of titles and abstracts from a given library-based systematic search. Our purpose is to evaluate and refine the LitQuest under real-world conditions to (1) estimate the efficiency of the LitQuest in terms of cost savings in human resources required for screening, (2) evaluate the performance of the LitQuest artificial intelligence algorithm using Work Saved over Sampling (WSS) for a certain recall, and (3) undertake human tests of the accuracy of the LitQuest by sampling rejected articles and checking that each is appropriately classified. Though these aims are tested through the LitQuest, the knowledge gains are intended to have broader application for others working in the AI-systematic review space.

## Method

### LitQuest Design and Workflow

The Living Review System (LitQuest version 1.0; Grbin et al., 2022) is a cloud-based web platform designed to allow researchers to leverage AI capability to facilitate timely and living reviews. Researchers can engage the system when starting a new review to achieve time savings in the reference screening phase of a review. They can also upload the results of an historical review (i.e. one in which all references have already been manually screened) to generate an algorithm that may be applied to new literature to maintain a living review. A web-based, menu-driven user interface was chosen in preference to a command-line, syntax-driven program to provide a more visually appealing interface and to reduce potential barriers in uptake due to limited coding experience (O’Connor et al., 2019).

Several features that are common to users of other citation management systems (e.g. Covidence) are included in the LitQuest to obviate the need for use of multiple programs to complete one’s review. Capacity is included for multiple raters to review the literature independently, and to then receive a report detailing articles for which disagreements arise. Further, data on duplicate removal, number of articles screened, and their allocation into ‘relevant’ versus ‘not relevant’ categories are stored for use for PRISMA flowcharts for the end-user.

New features currently under development (but not used in the present paper) include those designed to: (1) enhance confidence in the system (showing end users the keywords that are most important for the algorithm; with provision of opportunity for human input to help adjust/correct the algorithm), (2) enhance usefulness (e.g. text summarisation [within constraints of copyright rules] to concisely summarise key findings; text flagging capability to help with extraction of key data), and (3) allow the algorithm for one project to be accessible to other users to enhance generalizability of the algorithm through transfer learning as part of a broader commitment to open science and scientific progress.

The key steps in our LitQuest workflow are described below.

In Step 1 (*reference search/results upload*), researchers conduct a search across relevant databases, export results as an EndNote XML or.RIS file and upload this to the LitQuest for de-duplication. As is standard for this stage of screening in a manual review, imported files contain title, author, abstract, and DOI (assisting with back and forward search) as inputs for screening.

In Step 2 (*reference screening*), the LitQuest presents articles individually on the screen for the end-user to rate. In traditional screening approaches, the rater manually evaluates all articles for possible inclusion in their intended review. In contrast, the LitQuest system utilises AI techniques (specifically, machine learning) to support researchers in determining whether a reference is relevant. This is enabled through the use of active learning, a technique whereby the machine learner (i.e. the algorithm) adapts its understanding of a set of data (the list of search results) with each example it is given (end-user determination of reference relevance). In this way, the LitQuest can be considered a supervised machine learning model, (i.e. a model that learns by example) that can be trained to screen search results in partnership with human reviewers. This partnership is reflected in the description of the system as semi-automated. Recent work shows that an active learner can identify relevant, unsorted articles more quickly than other contemporary AI approaches (Yu et al., 2018).

By default, the LitQuest requires end-users to manually screen 10 relevant and 10 not relevant articles before generating the first iteration of an algorithm to sort the remaining articles into ‘relevant’ and ‘not relevant’ categories. This initial algorithm is refined through the active learner, and given a value between 0 and 1, which allows the LitQuest to simply present references in descending score order of relevance. Relevancy of manually sorted articles is displayed visually, with relevant articles coded as green with two ticks and not relevant coded as red with a dash. In cases where multiple raters independently screen references, discrepancies in assessment of an article are flagged by the LitQuest for users to reconcile. Discrepant ratings do not enter into the algorithm, and hence the algorithm does not diverge for multiple raters.

In Step 3 (*stopping rules*), LitQuest recommends continued manual screening of references sorted by the active learning model until the stopping criteria are met, which is triggered after a streak of 40 papers that are not relevant, and with relevancy confidence scores of < 0.5 for all remaining references. At this point the LitQuest AI algorithm is programmed to stop, as it is unlikely that many more relevant papers will be found and, thus, the user is prompted to stop screening. The LitQuest then asks if the user would like to mark all remaining unscreened references as “not relevant” to complete the screening phase of the review. It is important to note that the LitQuest default stopping rule is only a guide and can be overridden by simply ignoring the recommendation to stop. The LitQuest stopping rule may also be subject to change in future versions of the LitQuest as more data from end-user testing allows for evaluation and comparison of performance of different stopping rules. We also note though that, at present, a consensus view is lacking in the literature with respect to the best approach for a stopping rule (Callaghan & Müller-Hansen, 2020).

In Step 4 (*full-text screening and review*), on completion of screening, the LitQuest produces a list sorting all references into ‘relevant’ versus ‘not relevant’ for full-text screening. PDFs are collated for full-text screening and data extraction. The LitQuest has functionality to attach PDFs individually to relevant citations. It also supports bulk uploading of PDFs. To do this, relevant citations are exported as an RIS file from the LitQuest. This RIS file is then imported into EndNote so that PDFs can be located using EndNote’s automated ‘Find Full Texts’ function. Once located, the citations and PDFs are exported from EndNote and imported into the LitQuest for full-text screening.

It is important to emphasise that the LitQuest does not currently perform automatic text extraction because (1) a model would need to be fine-tuned and calibrated to ensure reliable summarisation of the article contents, lest the summarisation be misleading in casting a vote for inclusion, and (2) uploading non creative commons articles to OpenAI API for summarisation may represent a breach of the access and usage conditions for the material. In the future, LitQuest may be able to overcome this by using an open source model that is self-hosted as part of LitQuest, accompanied with specific legal terms governing the use of it in conjunction with the LitQuest platform.

LitQuest is currently under IP and commercial development. To discuss access to LitQuest, please contact the corresponding author.

### LitQuest Evaluation

Evaluation of the efficiency of the LitQuest was benchmarked using three primary outcome measures assessing LitQuest efficiency, performance, and accuracy. Data on each of these measures has been drawn from a set of seven systematic reviews, comprising a review series on predictors, natural history and consequences of early patterns of relational health within family systems, commissioned by the Paul Ramsay Foundation, Australia. Reviews were based on comprehensive, library-based, systematic searches of the global literature on early relational health, using gold-standard database searching techniques and accessing literature across all major platforms/databases: PubMed, MedLine, PsycInfo, and EBSCOBase. Title and abstract screening was conducted within the LitQuest, overseen by a review series program manager and completed by small teams of trained research assistants.

**LitQuest Efficiency** was estimated in terms of cost savings in human resources required for screening. Routinely collected meta-data on the number of articles screened in order to reach stop criteria were collected across each of the seven systematic searches, with the proportion needing human screening being calculated as the number of papers screened to stop criteria divided by the total number of articles to be screened (i.e. the total number of articles imported from the literature search after de-duplication). The mean proportion screened by the research team, including range of proportions screened across all reviews, was used to provide a marker of LitQuest efficiency.

**LitQuest Performance** was evaluated using Work Saved over Sampling (WSS) for a certain recall (in this case, 95% recall). This was calculated as the:

$$\left( {{\text{Number of Candidate Papers }}{-}{\text{Number of Manual Screenings}}} \right)/\left( {\text{Number of Candidate Papers}} \right) - \left( {{1}{-}{\text{Recall achieved}}} \right)$$

While other indicators (e.g. Balanced F-Score) are often employed for evaluation of model performance, these metrics fail to provide a holistic representation of rater preferences, and as such, provide a crude and unreliable approximation of performance of the model to benchmark in comparison to human raters. WSS is used for evaluation of other AI tools for systematic reviews (e.g. van de Schoot et al., 2021 who used a 95% recall threshold also).

**LitQuest Accuracy** was estimated in terms of the error rate in missing relevant articles after stop criteria had been reached. This was estimated by randomly sampling 100 articles from the full set of articles marked ‘not relevant’ by the LitQuest (and not screened by the research assistant team) after reaching the LitQuest stop criteria, and checking that each was appropriately classified. The mean proportion of misclassified articles flagged as ‘not relevant’ on completion of screening was used to provide a marker of LitQuest accuracy.

We note too that while LitQuest efficiency and LitQuest performance metrics are conceptually similar, it is possible that they may return different results. In particular, a stopping rule could be applied too early and thus miss relevant articles. The LitQuest performance metric thus provides some assurance about the stopping rule by quantifying the amount of titles and abstracts needing to be screened to identify a set percentage of relevant articles (in this case, 95%). Our LitQuest accuracy metric provides further test of appropriateness of the stopping rule.

## Results

Table 1 shows LitQuest efficiency per study in terms of the variability in proportions of articles that needed to be manually screened within the LitQuest by the research team to reach stopping criteria. The most facilitated review required 23% of titles and abstracts to be manually screened. The least facilitated review required all articles to be reviewed because the LitQuest stop criteria was not reached. On average, across all seven reviews, 45% of titles and abstracts needed to be manually screened within the system. This dropped to 36% when the one outlier requiring all articles to be screened was removed from the average. Taken together, these findings translate to considerable saving of time, human resource, and cost for screening phase of a systematic review.

**Table 1:** Summary of the efficiency of the LitQuest in expediating screening of titles and abstracts from a systematic search of library databases

Review	References	Review topic	Total articles	Number screened	Percent screened	Number unscreened
1	O’Dean et al. (2025)	Relational ecology of early child development	10,059	3531	35.1	6528
2a	Zhang et al. (2025)	Early RH and brain development	4915	4915	100.0	0
2b	Painter et al. (2025)	Early RH and relational development	15,454	3364	21.8	12,090
3	Fuller-Tyszkiewicz et al. (2025)	Early childhood RH interventions	4317	1980	45.9	2337
4	McNeair et al. (2024)	Australian indigenous RH knowledge	5141	2739	53.3	2402
5	Macdonald et al. (2025)	Intergenerational foundations of early RH	11,336	4220	37.2	7116
6	Allen et al. (2025)	Child, adolescent and young adult RH interventions	2366	557	23.5	1809
		Total	53,588	21,306	n/a	32,282
		Average	7655	3044	45.3	4612

MedLine, PsycINFO, and Embase. LitQuest stopping ruled defined by a manual screening streak of 40 irrelevant articles with < 50% probability of remaining articles being relevant

*RH* relational health

Table 2 shows the performance of the LitQuest artificial intelligence algorithm using work saved over sampling (WSS) for a 95% recall. The greatest WSS was 73% which means that to recall 95% of relevant articles in an overall set of articles, we needed to screen 27% of the total set. The smallest WSS was 42%, which means that to recall 95% of relevant articles, we needed to screen 58% of total articles. Overall, average work saved was estimated to be 59%; thus, less than half of the titles and abstracts (41%) needed to be screened to identify relevant literature.

**Table 2:** Summary of worked saved over sampling (WSS) post exceeding the stopping rule

Review	References		Review topic	Total articles	Number screened	Number relevant	WSS (percentage)
1	O’Dean et al. (2025)		The social ecology of ERH	10,059	3531	452	60
2a	Zhang et al. (2025)		ERH and brain development	4915	4915	52	n/a
2b	Painter et al. (2025)		ERH and relational development	15,454	3364	242	73
3	Fuller-Tyszkiewicz et al. (2025)		Early childhood ERH interventions	4317	1980	463	49
4	McNeair et al. (2024)		Indigenous RH knowledge	5141	2739	61	42
5	Macdonald et al. (2025)		Intergenerational foundations	11,336	4220	56	57
6	Allen et al. (2025)		Intergenerational interventions	2366	557	138	71
			Total		21,306	1464	
			Average		3044	209	59

LitQuest stopping ruled defined by a manual screening streak of 40 irrelevant articles with < 50% probability of remaining articles being relevant

*WSS* work saved over sampling, *RH* relational health

Table 3 shows variability in accuracy of assignment of titles and abstracts to ‘relevant’ and ‘not relevant’ on reaching the LitQuest stopping criteria. On average, across all seven reviews, 0% of titles and abstracts were incorrectly assigned indicating an exceedingly high level of accuracy in discriminating relevant from not relevant articles after human training.

**Table 3:** Summary of the accuracy of the LitQuest as determined by percent misclassified as irrelevant post exceeding the stopping rule

Review	References		Review topic	Number unscreened	Number randomly sampled	Number relevant or unsure
1	O’Dean et al. (2025)		The social ecology of ERH	6528	100	0
2a	Zhang et al. (2025)		ERH and brain development	0	n/a	
2b	Painter et al. (2025)		ERH and relational development	12,090	Not conducted	
3	Fuller-Tyszkiewicz et al. (2025)		Early childhood ERH interventions	2337	100	0
4	McNeair et al. (2024)		Indigenous RH knowledge	2402	100	0
5	Macdonald et al. (2025)		Intergenerational foundations	7116	100*	0
6	Allen et al. (2025)		Intergenerational interventions	1809	Not conducted	
		Total		32,282	300	0
		Average		4612	100	0

LitQuest stopping ruled defined by a manual screening streak of 40 irrelevant articles with < 50% probability of remaining articles being relevant. Articles for screening were divided among 4 screeners who had worked on the review and were familiar with eligibility criteria (single screening applied)

*RH* relational health

Title-abstract screening of randomly selected articles from remaining corpus after LitQuest stop signal activated (i.e. LitQuest screened at ‘not relevant’). Where * indicated, the articles screened were drawn from the remaining corpus without random selection, i.e. were those with the highest LitQuest-assigned confidence rating

From an applied perspective, across all seven reviews included in this evaluation of the LitQuest, our experience was that reviewers screened around 50 title-abstracts per hour. If we assume 50/hr then for a 10,000 article review, we would need 10,000 × 2/50 = 400 h screening time between two reviewers. If only 36% were manually screened (average cited above without the outlier), there would be a saving of 256 h work, that is almost 7 weeks of full-time work (assuming a 37.5 h work week).

## Discussion

Here we provide evidence of the efficiency, performance and accuracy of a new AI-based title and abstract screening tool, which is the first component of a broader Living Review System (LitQuest) our team is currently developing. We show that use of the LitQuest for title and abstract screening for a set of reviews (1) reduced review time costs by more than 55%, (2) quickly learnt from human user input, and (3) very accurately discerned relevant from not relevant articles beyond a conservative stopping algorithm. These performance statistics suggest that AI-based analysis of textual data from article abstracts has the potential to yield considerable time savings in human contribution and accurate prediction of article relevance (e.g. O’Mara-Eves et al., 2015).

LitQuest performance statistics are consistent with other AI-based screening tools in showing considerable time savings from AI augmented abstract screening (Rathbone et al., 2015; Tsou et al., 2020; van de Schoot et al., 2021). We also note differences in the proportion of articles needing to be manually screened across reviews in our study, which is also found in other performance evaluations for existing AI tools for screening of articles. For the one review where all articles were manually screened, it is possible that lexical heterogeneity may have made it more difficult for the AI algorithm to converge and suggest a stop point. In such a case, the conservative nature of LitQuest may be warranted to avoid missing relevant articles. For all other reviews in this series, there was no clear pattern linking the proportion of articles needing to be screened to the proportion of relevant articles within the overall set of articles (relevant proportion ranged from 0.5 to 11%). However, there was a trend for the AI algorithm to reduce workload to a greater extent in larger reviews (i.e. with more articles in the overall set). It is thus likely that the algorithm’s accuracy improves with more data. Two preliminary recommendations may be drawn from this: (1) value gained from AI may be less with small pools of articles, however (2) greatest time saving may occur with large numbers of articles, precisely the context where AI uplift is most needed.

Despite encouraging findings from the present study, several limitations offer viable pathways for further research exploration. First, we evaluated LitQuest performance across a range of reviews that had differing research foci. Even so, they all had relational health as a common topic, and methodologies were either prospective cohort designs or randomised control trials (RCTs). As design type was important for inclusion/exclusion criteria into these reviews, it is possible that positive performance of the LitQuest here reflects consistency in terminology for these research design types. Second, and related to the first point, more systematic evaluation of performance across disparate designs and a broader range of research disciplines is needed to better understand variability in performance as a function of review topic. Third, more detailed exploration of keywords used in underlying AI models to discriminate between relevant and not relevant articles may also be illuminating about which elements of these abstracts contribute to model performance. Finally, stability of models over time remains an area in need of further research attention. With the passage of time, key concepts and methodologies may change, rendering key terms in one’s original AI-based screening algorithm less effective for newer articles.

Despite growing enthusiasm for AI-based assistance for conducting reviews of the scientific literature across multiple knowledge domains, uses of artificial intelligence remain a nascent area of inquiry (Jonnalagadda et al., 2015; van Dinter et al., 2021). Further performance testing of the LitQuest is needed; however, findings presented here are encouraging and suggest an important role for AI-based screening software in the life cycle of a Living Systematic Review. Furthermore, the LitQuest is the first build in a broader digital ecosystem that will include the application of generative AI for full-text data extraction and thematic analysis, and export of synthesised knowledge to community-based web-portals to ensure open translation of aggregate research results. These broader ambitions, however, need to happen within the context of emerging permissions around the appropriate re-use of publisher copyright papers for scientific purposes.
